# Frequency of Urinary Tract Infections in Type 2 Diabetic Patients Taking Dapagliflozin

**DOI:** 10.7759/cureus.21720

**Published:** 2022-01-29

**Authors:** Sarfaraz Khan, Mubashar Sultan Hashmi, Muhammad A Rana, Ghulam Mujtaba Zafar, Sadia Asif, Muhammad Talha Farooq, Sarmad Zahoor

**Affiliations:** 1 Internal Medicine/Endocrinology, Hayatabad Medical Complex, Peshawar, PAK; 2 Internal and Critical Care Medicine, Bahria International Hospital, Lahore, PAK; 3 Critical Care Medicine, Bahria International Hospital, Lahore, PAK; 4 Urology, The Children's Hospital, Lahore, PAK; 5 Rheumatology, Fatima Memorial Hospital, Lahore, PAK; 6 Internal Medicine, Fatima Memorial Hospital, Lahore, PAK; 7 Cardiovascular Medicine, Punjab Institute of Cardiology, Lahore, PAK

**Keywords:** type 2 diabetes mellitus, sodium glucose co-transporter 2 inhibitors (sglt2i), dapagliflozin, glycosuria, urinary tract infection

## Abstract

Background

Urinary tract infections (UTIs) are common in patients with diabetes. The use of sodium-glucose cotransporter-2 inhibitors (SGLT2i) to achieve good glycemic control increases glucose levels in urine. This glycosuria further enhances the risk of UTIs. This study aimed to evaluate the frequency of UTIs in patients with type 2 diabetes receiving the SGLT2i dapagliflozin as an add-on therapy.

Methods

We conducted this cross-sectional study at the Endocrinology Department of Hayatabad Medical Complex in Peshawar from April 2020 to September 2020. A total of 400 patients with diabetes receiving either 5 mg or 10 mg of dapagliflozin as an add-on therapy for the treatment of type 2 diabetes were included in this study. We collected blood and urine samples from participants and measured glycosylated hemoglobin levels. Urine samples were cultured on cysteine lactose electrolyte deficient agar. We used IBM SPSS Statistics for Windows, version 25.0 (IBM Corp., Armonk, NY) to analyze our data.

Results

The prevalence of UTIs in diabetic patients receiving 5 mg or 10 mg of dapagliflozin was 5.3%. Women were more affected (76.2%) than men (p < 0.05). UTIs were more prevalent in patients older than 50 years (85.7%) than in any other age group. The dose strength of dapagliflozin was not associated with UTIs (p > 0.05).

Conclusion

This study examined UTIs in patients taking dapagliflozin for the treatment of type 2 diabetes. These infections were mild to moderate and were treated easily. None of these infections caused the patient to discontinue the treatment. Dapagliflozin is well-tolerated in patients with diabetes but should be used with appropriate caution and monitoring.

## Introduction

Type 2 diabetes (T2D) is a global problem affecting millions of people. The number of patients with T2D increased from 121 million to 285 million from 2000 to 2010 [1,2]. Approximately 463 million adults live with diabetes, but this is projected to increase to 578 million by 2030 and 700 million by 2045. T2D accounts for 90% of all types of diabetes [1].

Typical therapeutic options for treating T2D include metformin, sulfonylurea, sitagliptin, glucose-like peptide-1 receptor agonists, and insulin. Sodium-glucose cotransporter-2 inhibitors (SGLT2i) are recent therapeutic agents for treating T2D that work in the kidneys [3,4]. Glucose is reabsorbed in the kidneys in the proximal tubule of the loop of Henle. The transporter protein responsible for this reabsorption is sodium-glucose cotransporter-2 (SGLT2), composed of 672 amino acids located in the S1 segment of the proximal renal tubule. This is the site of action of SGLT2i drugs like empagliflozin, dapagliflozin, and canagliflozin [4,5]. Dapagliflozin has been extensively studied in diabetic patients in controlled clinical trials either alone or in combination with other antihyperglycemic agents and was approved by the US Food and Drug Administration as a treatment option for T2D [6,7].

Increased urinary glucose excretion can lead to urinary tract infections (UTIs) [8]. The possible mechanism is that high glucose levels provide a good environment for bacterial proliferation. One study reported that *Escherichia coli* increased in proportion with glucose levels [9]. Our study aimed to determine the frequency of UTIs in patients with T2D receiving dapagliflozin (in 5 or 10 mg doses).

## Materials and methods

We conducted a prospective, observational, cross-sectional study from April 2020 to September 2020 in Hayatabad Medical Complex (HMC), a tertiary care hospital in Peshawar, Khyber Pakhtunkhwa. We calculated the sample size of 400 patients using the 50% prevalence rate. The prevalence of UTIs was assumed to be 50% in T2D patients receiving dapagliflozin because of the lack of data in our region [10]. We used non-probability consecutive sampling to enlist study participants.

All T2D patients aged 30-80 years using dapagliflozin at a dose of 5 mg or 10 mg as an add-on therapy to oral antihyperglycemic agents or insulin were included in the study. We excluded any T2D patients with culture-proven UTIs within one month of the start of dapagliflozin, currently catheterized patients, or those with a history of UTI in two weeks prior to the start of the study. We also excluded chronic kidney disease patients with estimated glomerular filtration rates of <45 mL/min, patients on renal replacement therapy, and patients who received renal transplants. A standard questionnaire was designed for recording patients' history and demographic data.

The study was conducted after approval from the ethical and research committee of the hospital. The patients were advised to collect midstream urine in a sterile urine bottle for microbiological analyses. The urine samples were cultured in the microbiology laboratory of HMC. Cysteine lactose electrolyte deficient agar was used for culturing the urine. The plates were correctly incubated at 37ºC for 24 hours. We also measured patients’ serum levels of glycated hemoglobin (HbA1c).

We used IBM SPSS Statistics for Windows, version 25.0 (IBM Corp., Armonk, NY) to analyze our data. Results were considered statistically significant with p-values < 0.05. Patients were assigned into two age groups: those aged 30 to 50 years and those older than 50 years. Chi-square was used to find an association between the groups post-stratification.

## Results

**Figure 1:**
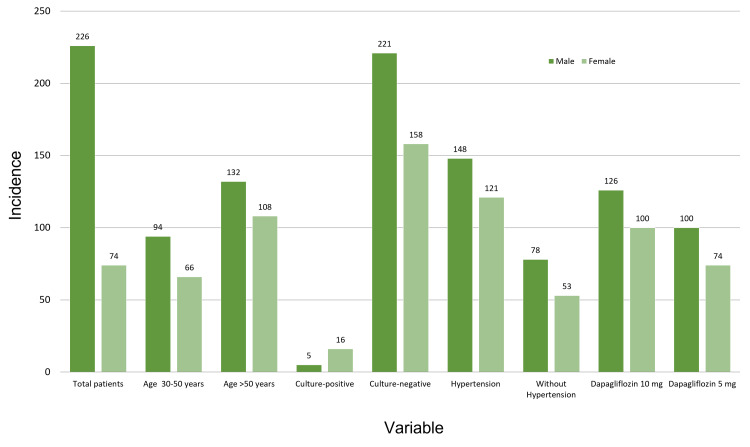
Gender-based distribution of frequencies by variables

A total of 400 patients comprised our study population with a male to female ratio of 1.29:1 (226 men, 56.5%; 174 women, 43.5%). The mean age of our study participants was 55.2 ± 6.2 years, and the mean HbA1c was 8.06% ± 0.52%. A total of 131 patients (32.8%) had hypertension concurrent with diabetes. A total of 174 (43.5%) patients received 5 mg dapagliflozin and 226 (56.5%) patients received 10 mg dapagliflozin. A small percentage of our study population had UTIs (n = 21; 5.3%). Table 1 presents the frequencies of all the categorical variables. Figure 1 shows the frequencies of the variables based on gender.

**Table 1:** Frequencies of all the categorical variables

Variables		Frequencies, n (%)
Age strata (years)	30-50	160 (40%)
	>50	240 (60%)
Gender	Male	226 (56.5%)
	Female	174 (43.5%)
Hypertension	Yes	131 (32.8%)
	No	269 (67.2%)
Dosage of dapagliflozin	5 mg	174 (43.5%)
	10 mg	226 (56.5%)
UTI symptoms	Present	29 (7.3%)
	Absent	371 (92.7%)
Culture/UTIs	Positive/present	21 (5.3%)
	Negative/absent	379 (94.7%)

We noted significantly more confirmed UTI cases in women (n = 21) than in men (n = 5; p < 0.05). Patients older than 50 years had a significantly higher prevalence of UTIs than patients aged 30 to 50 years (p < 0.05). The dosage of the drug was not significantly associated with UTIs. Table 2 provides the results of other variables in association with UTIs.

**Table 2:** Association of different variables with UTIs

Variables		Urine cultures/UTIs		P-value
		Positive/present (n)	Negative/absent (n)	
Age strata	30-50 years (n = 160)	3	157	0.01
	>50 years (n = 240)	18	222	
Gender	Male (n = 226)	5	221	0.002
	Female (n = 174)	16	158	
Hypertension	Yes (n = 131)	9	122	0.31
	No (n = 269)	12	257	
Dapagliflozin dose	5 mg (n = 174)	9	165	0.95
	10 mg (n = 226)	12	214	
UTI symptoms	Present (n = 29)	20	9	<0.001
	Absent (n = 371)	1	370	

## Discussion

SGLT2i are a relatively new class of antihyperglycemic drugs, and research suggests that they are associated with a high risk of UTIs [8]. SGLT2i agents such as dapagliflozin increase the concentration of glucose in the urine, providing an ideal environment for colonization and growth of bacteria, which contribute to the onset of UTIs [10]. Our study aimed to assess UTI frequency in T2D patients treated with two different dose sizes of SGLT2i. While our results indicated a risk of UTI in these patients associated with SGLT2i uses, the severity of the UTIs was mild to moderate and could be treated easily. None of the UTIs caused discontinuation of treatment by SGLT2i among the patients.

In our study, 5.3% of T2D patients receiving dapagliflozin at a dose of either 5 mg or 10 mg were diagnosed with UTIs. Previous research reported a 4% to 9% prevalence of UTIs in clinical trials with a difference of only 1% to 1.5% higher than placebo [11-13]. Another study reported a low prevalence of UTIs in T2D patients receiving dapagliflozin as monotherapy [14]. Our study had the highest UTI prevalence in patients older than 50 years, especially among women. This observation is consistent with previous reports [4,11,12] and might be due to the postmenopausal changes in the female urogenital system [15].

A higher dose of dapagliflozin increases urinary excretion of glucose and would theoretically increase the risk of UTIs by providing a good flourishing environment for the bacteria. This increase in risk was supported by the results of a recent report [4]. However, we did not observe this in our study, as UTIs were not statistically associated with the dose strength of dapagliflozin (p = 0.95). Our findings align with those of another study, which reported that with a higher SGLT2i dose, glycosuria increased but did not affect the incidence of UTIs [16].

Dapagliflozin reduces the amount of glucose in the blood by inhibiting reabsorption in the kidneys [17]. The literature presents conflicting data on the association of UTIs with dapagliflozin dose size [4,16,17]. It is important to identify confounding variables in these studies, including ours. For example, a history of repeated UTIs can predispose a patient to future UTIs, as seen in the placebo group in certain studies [18]. These confounding factors limit our ability to generalize our results to broader populations. This limitation also holds when determining association [12]. According to the published data, SGLT2i use is more associated with genital infections than UTIs [19]. In our study, more women were affected by UTIs than men, aligning with previous reports [16,20].

There were a few limitations to our study. It was a single-center study involving people of the same ethnicity. Also, we did not assess the impact of different doses of dapagliflozin on glycosuria. Further studies with improved design could better explain the relationship between glycosuria at different doses of SGLT2I treatments and UTI prevalence.

## Conclusions

A significant percentage of UTIs was observed in patients using dapagliflozin for the treatment of T2D. These infections were mild to moderate and treated easily, and none caused the patient to discontinue the use of SGLT2i. Thus, dapagliflozin is a safe treatment option for patients with T2D if used with the appropriate levels of caution and monitoring.

## References

[1] Geerlings SE (2008). Urinary tract infections in patients with diabetes mellitus: epidemiology, pathogenesis and treatment. Int J Antimicrob Agents.

[2] Zheng Y, Ley SH, Hu FB (2018). Global aetiology and epidemiology of type 2 diabetes mellitus and its complications. Nat Rev Endocrinol.

[3] Chamberlain JJ, Herman WH, Leal S, Rhinehart AS, Shubrook JH, Skolnik N, Kalyani RR (2017). Pharmacologic therapy for type 2 diabetes: synopsis of the 2017 American Diabetes Association Standards of Medical Care in Diabetes. Ann Intern Med.

[4] Hsia DS, Grove O, Cefalu WT (2017). An update on sodium-glucose co-transporter-2 inhibitors for the treatment of diabetes mellitus. Curr Opin Endocrinol Diabetes Obes.

[5] Heald AH, Fryer AA, Anderson SG (2018). Sodium-glucose co-transporter-2 inhibitors, the latest residents on the block: impact on glycaemic control at a general practice level in England. Diabetes Obes Metab.

[6] Lupsa BC, Inzucchi SE (2018). Use of SGLT2 inhibitors in type 2 diabetes: weighing the risks and benefits. Diabetologia.

[7] Plosker GL (2014). Dapagliflozin: a review of its use in patients with type 2 diabetes. Drugs.

[8] Liu J, Li L, Li S, Jia P, Deng K, Chen W, Sun X (2017). Effects of SGLT2 inhibitors on UTIs and genital infections in type 2 diabetes mellitus: a systematic review and meta-analysis. Sci Rep.

[9] Geerlings SE, Brouwer EC, Gaastra W, Verhoef J, Hoepelman AI (1999). Effect of glucose and pH on uropathogenic and non-uropathogenic Escherichia coli: studies with urine from diabetic and non-diabetic individuals. J Med Microbiol.

[10] Mama M, Manilal A, Gezmu T, Kidanewold A, Gosa F, Gebresilasie A (2019). Prevalence and associated factors of urinary tract infections among diabetic patients in Arba Minch Hospital, Arba Minch province, South Ethiopia. Turk J Urol.

[11] Haas B, Eckstein N, Pfeifer V, Mayer P, Hass MD (2014). Efficacy, safety and regulatory status of SGLT2 inhibitors: focus on canagliflozin. Nutr Diabetes.

[12] Johnston R, Uthman O, Cummins E (2018). Corrigendum: canagliflozin, dapagliflozin and empagliflozin monotherapy for treating type 2 diabetes: systematic review and economic evaluation. Health Technol Assess.

[13] Luzon E, Blake K, Cole S, Nordmark A, Versantvoort C, Berglund EG (2017). Physiologically based pharmacokinetic modeling in regulatory decision-making at the European Medicines Agency. Clin Pharmacol Ther.

[14] Wiviott SD, Raz I, Bonaca MP (2019). Dapagliflozin and cardiovascular outcomes in type 2 diabetes. N Engl J Med.

[15] Foxman B (1999). Urinary tract infection in postmenopausal women. Curr Infect Dis Rep.

[16] Johnsson KM, Ptaszynska A, Schmitz B, Sugg J, Parikh SJ, List JF (2013). Urinary tract infections in patients with diabetes treated with dapagliflozin. J Diabetes Complications.

[17] Misra M (2013). SGLT2 inhibitors: a promising new therapeutic option for treatment of type 2 diabetes mellitus. J Pharm Pharmacol.

[18] Storme O, Tirán Saucedo J, Garcia-Mora A, Dehesa-Dávila M, Naber KG (2019). Risk factors and predisposing conditions for urinary tract infection. Ther Adv Urol.

[19] Lega IC, Bronskill SE, Campitelli MA (2019). Sodium glucose cotransporter 2 inhibitors and risk of genital mycotic and urinary tract infection: a population-based study of older women and men with diabetes. Diabetes Obes Metab.

[20] Watts NB, Bilezikian JP, Usiskin K, Edwards R, Desai M, Law G, Meininger G (2016). Effects of canagliflozin on fracture risk in patients with type 2 diabetes mellitus. J Clin Endocrinol Metab.

